# The inhibition of chloride intracellular channel 1 enhances Ca^2+^ and reactive oxygen species signaling in A549 human lung cancer cells

**DOI:** 10.1038/s12276-019-0279-2

**Published:** 2019-07-17

**Authors:** Jae-Rin Lee, Jong-Yoon Lee, Hyun-Ji Kim, Myong-Joon Hahn, Jong-Sun Kang, Hana Cho

**Affiliations:** 1 Department of Molecular Cell Biology, Sungkyunkwan University, Suwon, Korea; 2 Single Cell Network Research Center, Sungkyunkwan University, Suwon, Korea; 3 Department of Physiology, Sungkyunkwan University, Suwon, Korea

**Keywords:** Ion channel signalling, Calcium signalling

## Abstract

Chloride intracellular channel 1 (CLIC1) is a promising therapeutic target in cancer due to its intrinsic characteristics; it is overexpressed in specific tumor types and its localization changes from cytosolic to surface membrane depending on activities and cell cycle progression. Ca^2+^ and reactive oxygen species (ROS) are critical signaling molecules that modulate diverse cellular functions, including cell death. In this study, we investigated the function of CLIC1 in Ca^2+^ and ROS signaling in A549 human lung cancer cells. Depletion of CLIC1 via shRNAs in A549 cells increased DNA double-strand breaks both under control conditions and under treatment with the putative anticancer agent chelerythrine, accompanied by a concomitant increase in the p-JNK level. CLIC1 knockdown greatly increased basal ROS levels, an effect prevented by BAPTA-AM, an intracellular calcium chelator. Intracellular Ca^2+^ measurements clearly showed that CLIC1 knockdown significantly increased chelerythrine-induced Ca^2+^ signaling as well as the basal Ca^2+^ level in A549 cells compared to these levels in control cells. Suppression of extracellular Ca^2+^ restored the basal Ca^2+^ level in CLIC1-knockdown A549 cells relative to that in control cells, implying that CLIC1 regulates [Ca^2+^]_i_ through Ca^2+^ entry across the plasma membrane. Consistent with this finding, the L-type Ca^2+^ channel (LTCC) blocker nifedipine reduced the basal Ca^2+^ level in CLIC1 knockdown cells to that in control cells. Taken together, our results demonstrate that CLIC1 knockdown induces an increase in the intracellular Ca^2+^ level via LTCC, which then triggers excessive ROS production and consequent JNK activation. Thus, CLIC1 is a key regulator of Ca^2+^ signaling in the control of cancer cell survival.

## Cancer: chloride transport protein protects tumors

A chloride channel, a membrane protein involved in chloride transport, indirectly regulates calcium signaling to protect cancer cells from free radicals and DNA damage. Hana Cho from Sungkyunkwan University School of Medicine in Suwon, South Korea, and colleagues inhibited the CLIC1 chloride channel in human lung cancer cells and observed increased activity of a calcium channel. This led to elevated levels of calcium ions within the cells, which in turn triggered the production of chemically reactive molecules that both damaged DNA and activated a signaling cascade that promotes cell death. Many different types of cancer express CLIC1 at high levels. The findings show how the chloride channel helps protect tumor cells from surges in calcium and free radicals, and highlight the therapeutic potential of targeting CLIC1 to slow tumor growth.

## Introduction

Recent studies have revealed the role of ion channels in the development of different cancers. Currently, Cl^-^ channels are considered the most active channels during tumorigenesis^1,2^. A high rate of proliferation, active migration, and invasiveness into nonneoplastic tissues are specific properties of neoplastic transformation. All these actions require partial or total involvement of Cl^-^ channel activity^3–6^. Thus, this class of membrane proteins could represent valuable therapeutic targets for the treatment of resistant tumors. However, drug design targeting ion channels is difficult because of the vital role of these channels for essential physiological functions in normal cells. Considering this difficulty, a new protein family, the chloride intracellular channels (CLICs)—particularly CLIC1—could be a promising class of therapeutic targets because of their intrinsic properties. First, CLIC1 is overexpressed in particular tumor types, such as hepatocellular carcinoma^7^, gallbladder carcinoma^8^, gastric carcinoma^9^, and colorectal cancer^10,11^. Second, CLIC1s change their localization from cytosolic to transmembrane as active ionic channels or signal transducers during cell cycle progression in certain cases^12,13^. These changes in intracellular localization and channel function, which are associated with malignant transformation, may offer a distinct target for cancer therapy that can likely spare normal cells. Therefore, understanding the role and underlying molecular mechanism of CLIC1 in cellular transformation is important for designing a therapeutic strategy.

Multiple studies have shown that CLIC1 plays crucial roles in controlling the cell cycle, apoptosis, proliferation, invasiveness, and metastasis^8–10,13–24^. Given that changes in the level of reactive oxygen species (ROS) are fundamental for cell cycle progression^25,26^ and cancer cell survival^27^, it was suggested that CLIC1 could regulate ROS production in cancer cells. In fact, the inhibition of CLIC1 channel activity by the CLIC ion channel blocker IAA94 reduces intracellular ROS production during hypoxia-reoxygenation treatment in LOVO cells, human colon adenocarcinoma cells, suggesting that CLIC1 sustains ROS levels^28^. However, CLIC1 can also function as a negative regulator of ROS in other tumors, since the depletion of CLIC1 using siRNA in human esophageal squamous cell carcinoma induced apoptosis via the JNK pathway, which is strongly associated with excessive ROS production^29,30^. Furthermore, the mechanism by which CLIC1 regulates ROS is currently unclear. In this study, we investigated the role of CLIC1 and its molecular mechanism in A549 human lung cancer cells. We found that depletion of CLIC1 with shRNA in A549 human lung cancer cells increased DNA double-strand breaks both under control conditions and under treatment with the putative anticancer agent chelerythrine, with a concomitant increase in p-JNK levels. Intracellular Ca^2+^ measurements revealed that CLIC1 knockdown in A549 cells induced an increase in both the basal Ca^2+^ level and chelerythrine-induced Ca^2+^ signaling. In addition, CLIC1 knockdown greatly increased basal ROS levels, an effect that was prevented by BAPTA-AM, an intracellular calcium chelator. The LTCC blocker nifedipine, as well as the suppression of extracellular Ca^2+^, restored the basal Ca^2+^ level in CLIC1-knockdown A549 cells to that in control cells. Taken together, our results demonstrate that CLIC1 knockdown induces an increase in the intracellular Ca^2+^ level via LTCC, which then triggers excessive ROS production and consequent JNK activation.

## Materials and methods

### Plasmid construction

The shRNA sequences CLIC1-knockdown 1 (5′- GATCCCCGGGAGTCACCTTCAATGTTACTTCAAGAGAGTAACATTGAAGGTGACTCCCTTTTTA-3′) and CLIC1-knockdown 2 (5′- GATCCCCGATGAAGGTGTCTCTCAGAGGTTCAAGAGACCTCTGAGAGACACCTTCATCTTTTTA-3′) were cloned into the pSuper.retro vector (Oligoengine, Seattle, WA, USA). CLIC1 cDNA was amplified from MEFs and inserted into pEGFP-C1 (Clontech, Palo Alto, CA, USA) at the *BglII* and *XhoI* sites.

### Generation of antibodies

GST-CLIC1 proteins were purified from *E. coli* and cleaved with thrombin to remove the GST domain and were then used to immunize BALB/c mice. Immunized splenocytes were fused with myeloma cells and selected with HAT medium. Cell culture medium from the cloned hybridomas was analyzed with ELISA to identify specific antibodies against CLIC1. The specificity of the antibodies was tested with other CLICs (CLIC2, 3, 4, and 5).

### Cell culture and transfection

A549 human lung carcinoma cells were maintained in RPMI 1640 medium containing 10% FBS. To establish the CLIC1knockdown cell line, pSuper.retro-scrambled shRNA or the pSuper.retro-CLIC1 KD1 or KD2 shRNA constructs were transfected into A549 cells using Lipofectamine (Thermo Scientific, Waltham, MA, USA) and selected with 0.3 µg/ml puromycin. Cell clones were screened for CLIC1 knockdown by immunoblot analysis. For assessing the subcellular localization of CLIC1, A549 cells were transfected with pEGFP-C1 or pEGFP-C1-CLIC1 plasmids using Effectene (Qiagen, Valencia, CA, USA). For transient knockdown of CLIC1, 50 nM noncoding region siRNA (sense: 5′-UUCUCCGAACGUGUCACGUUU-3′; antisense: 5′-ACGUGACACGUUCGGAGAAUU-3′) or siRNA against CLIC1 (sense: 5′-GGGAGUCACCUUCAAUGUUUU-3′; antisense: 5′-AACAUUGAAGGUGACUCCCUU-3′) was transfected into A549 cells using Lipofectamine RNAiMAX (Invitrogen, Carlsbad, CA, USA).

### Immunocytochemistry

A549 cells stably expressing pSuper.retro-scrambled shRNA or pSuper.retro-CLIC1 KD1 or KD2 shRNA were stimulated with 50 µM chelerythrine for 24 h. Cells were fixed with 4% paraformaldehyde and permeablized with 0.5% Triton X-100 in PBS. Samples were blocked with 5% BSA in PBS and stained with anti-pγH2AX (Ser140) (Invitrogen). FITC-conjugated goat anti-mouse IgG (Jackson Laboratory, Bar Harbor, Maine, USA) secondary antibodies were used. For nuclear staining, Hoechst 33258 was used, and slides were mounted with ProLong Gold antifade mount (Thermo Scientific). Confocal images were obtained using an LSM 710 (Zeiss, Oberkochen, Germany).

### Immunoblot analysis

Cells were lysed on ice for 30 min in lysis buffer (50 mM Tris-HCl (pH 8.0) 0.1% Triton X-100, 50 mM sodium fluoride, 5 mM sodium pyrophosphate, 1 mM PMSF, 1 mM sodium orthovanadate, and 2 mM leupeptin). After centrifugation, the protein concentration in the supernatant was determined by a BSA kit (Pierce, Rockford, IL, USA). Samples were separated by SDS-PAGE and transferred and were then immunoblotted with the following antibodies: p-p38 MAPK (Thr180/Tyr182), p38 MAPK, p-SAPK/JNK (Thr183/Tyr185), SAPK/JNK, and p-Akt (Ser473) from Cell Signaling Technology (Beverly, MA, USA) and α-tubulin from Sigma-Aldrich (St. Louis, MO, USA).

### Solutions and drugs

The normal Tyrode’s (NT) solution contained (in mM) NaCl (143), KCl (5.4), CaCl_2_ (1.8), MgCl_2_ (0.5), NaH_2_PO_4_ (0.5), glucose (11.1), and HEPES (5) and was adjusted to pH 7.4 with NaOH. To make the Ca^2+^-free NT solutions, CaCl_2_ was replaced with equimolar MgCl_2_. Fura 2-AM was obtained from Thermo Scientific, and chelerythrine chloride and bisindolylmaleimide I were obtained from Tocris Bioscience (Bristol, UK). All other drugs were purchased from Sigma-Aldrich. Stock solutions of the drugs were made by dissolution in deionized water or DMSO according to the manufacturer’s specifications and were stored at −20 °C. On the day of the experiment, one aliquot was thawed and used. The final concentration of DMSO in the solutions was maintained below 0.1%.

### Reactive oxygen species (ROS) generation assay

For the measurement of intracellular ROS levels, the general ROS marker CM-H2DCFDA (Thermo Scientific) was used. Cells were incubated with 20 µM CM-H2DCFDA for 1 h and were then washed with PBS. CM-H2DCFDA fluorescence was measured using confocal laser scanning microscopy.

### [Ca^2+^]_i_ measurements

Cells were incubated with 3 µM Fura-2 AM (Life Technologies, Carlsbad, CA, USA) for 45 min in NT solution or Ca^2+^-free NT solution at room temperature. For fluorescence excitation, we used a polychromatic light source (xenon lamp-based, Polychrome-IV; TILL-Photonics), which was coupled to the epi-illumination port of an inverted microscope (IX70, Olympus, Tokyo, Japan) via a quartz light guide and a UV condenser. Fluorescence intensity was measured via a 40 × objective (Olympus), a charge-coupled device image intensifier camera (Andor Technology, Belfast, UK) and Metafluor software (Molecular Devices, Sunnyvale, CA, USA). Dual excitation at 340/380 was used with a 400-nm dichroic mirror, and emitted light was collected with a 450-nm long-pass filter.

### Statistical analysis

Data are presented as the means ± standard errors of the mean. Student’s *t-*test or one-way ANOVA was used to test for significance; *P* *<* 0.05 was considered statistically significant.

## Results

### CLIC1 knockdown exacerbated the cellular stress response in A549 cells

**Fig. 1:**
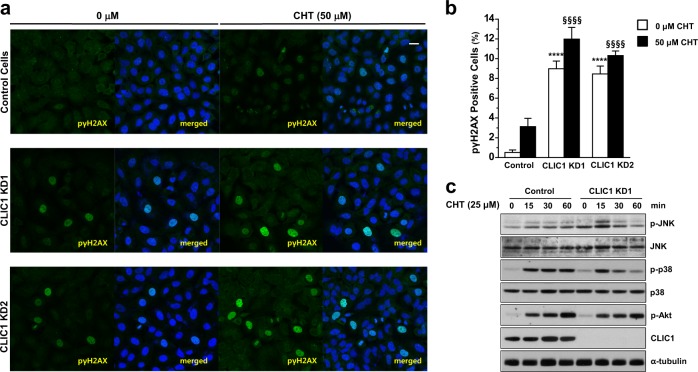
CLIC1 knockdown exacerbated the cellular stress response in A549 cells. **a** A549 cells were stimulated with vehicle or 50 μM chelerythrine for 24 h. Representative images showing pγH2AX (Ser140) (green) and Hoechst-stained nuclei (blue). Scale bar, 20 μm for all images. **b** Quantification of pγH2AX (Ser140)-positive cells. At least 400–900 cells were counted for each condition (Supplementary Table S1). ****P *<* 0.0001 (control vs CLIC1 KD with vehicle) or ^§§§§^*P* *<* 0.0001 (control vs CLIC1 KD with chelerythrine stimulation) by ANOVA with Tukey’s test (*P* < 0.05). **c** Depletion of CLIC1 activated MAPK differentially under ROS exposure. A549 cells were stimulated with 25 μM chelerythrine for the indicated times. The protein levels of mitogen-activated protein kinases (MAPKs), including JNK and p38, Akt and CLIC1, were determined by immunoblot analysis. Data from three independent experiments are shown

First, we investigated the role of CLIC1 in the regulation of the cellular stress response. To do this, we assessed the effects of CLIC1 knockdown on DNA damage in A549 cells. Immunostaining for the level of pγH2AX, a DNA double-strand break marker, in control and CLIC1- knockdown A549 cells revealed that CLIC1 knockdown significantly increased the level of pγH2AX (Fig. 1a). It is well known that a putative anticancer agent, chelerythrine, induces cellular stress in cancer cells^31^. Thus, we examined the effects of CLIC1 knockdown on chelerythrine-induced cellular stress in A549 cells. Consistent with the results of previous studies^31^, treatment with chelerythrine (50 µM) for 24 h increased the level of pγH2AX in control A549 cells, which was further elevated by CLIC1 knockdown (Fig. 1a, b). We used two different shRNAs for CLIC1 depletion and found that both shRNAs effectively reduced the level of CLIC1 protein (Supplementary Figure S1) and increased DNA double-strand breaks under both control and chelerythrine treatment conditions (Fig. 1a, b). In addition, chelerythrine did not alter the cellular localization of CLIC1-eGFP in A549 cells (Supplementary Figure S2).

It has been demonstrated that chelerythrine induces cellular stress and apoptotic cell death through mitogen-activated protein kinases (MAPKs), including c-Jun N-terminal kinase (JNK), p38, and Akt^32,33^. Thus, we examined the activation of JNK, p38, and Akt in the response to chelerythrine in CLIC1-knockdown A549 cells (Fig. 1c). CLIC1 knockdown resulted in elevated levels of the active, phosphorylated form of JNK (p-JNK), which was further increased by chelerythrine, compared to those in control cells. Interestingly, CLIC1-knockdown A549 cells exhibited a transient increase in the p-JNK level 15 min after chelerythrine treatment, while control cells showed a sustained increase. Thus, CLIC1 knockdown increased basal JNK activity and induced a surge in JNK activity upon chelerythrine treatment, which could function in tandem with efficient apoptotic machinery. In contrast, the levels of the active, phosphorylated forms of p38 (p-p38) and Akt (p-Akt) were unaltered in CLIC1-knockdown A549 cells (Fig. 1c). Both control and CLIC1- knockdown A549 cells exhibited strong activation of Akt starting 15 min after chelerythrine treatment. Interestingly, however, CLIC1 knockdown induced transient activation of p38 15 min after chelerythrine treatment, while control cells exhibited persistent activation. Currently, the basis of this transient activation of JNK and p38 in CLIC1- knockdown cells in response to chelerythrine treatment is unclear. However, since CLIC1 knockdown alone increased the p-JNK level, which was further elevated by chelerythrine treatment, JNK is an important mediator of the enhanced susceptibility of CLIC1- knockdown A549 cells to chelerythrine treatment.

### CLIC1 knockdown increased ROS in A549 cells

**Fig. 2:**
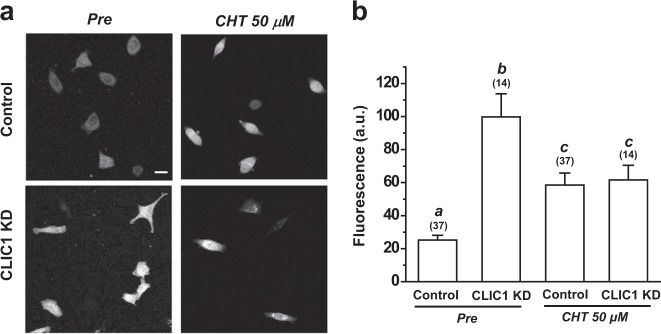
The basal level of ROS generation was increased in CLIC1-knockdown cells. **a** Representative images of staining for the ROS marker CM-H2DCFDA in control (upper panel) and CLIC1 KD cells (lower panel) before and after chelerythrine treatment (50 μM, 10 min). Scale bars, 20 μm. **b** CM-H2DCFDA fluorescence intensity in control and CLIC1 KD cells. The data are the means ± SEMs. The letters indicate statistically distinct groups (ANOVA with Tukey’s test, *P* < 0.05)

Whether CLIC1 knockdown increases ROS production in A549 cells was examined by using a CM-H2DCFDA probe. As shown in Fig. 2, CLIC1 knockdown via CLIC1 shRNA 1 increased the ROS level by ~4-fold in A549 cells. We then examined the effects of chelerythrine on ROS generation in control A549 cells and in cells transfected with CLIC1 shRNA 1. Consistent with the results of previous studies^31^, treatment with 50 μM chelerythrine increased the generation of ROS by 132.09% in control cells. The ROS level in CLIC1knockdown A549 cells was not further increased but rather decreased by 50 μM chelerythrine. However, the ROS level was still higher than that in untreated control A549 cells and was comparable to that in chelerythrine-treated control A549 cells (Fig. 2a, b). These data suggest that CLIC1 knockdown increased the ROS level in A549 cells.

### CLIC1 knockdown increased the basal Ca^2+^ level and augmented the effects of chelerythrine on [Ca^2+^]_i_

**Fig. 3:**
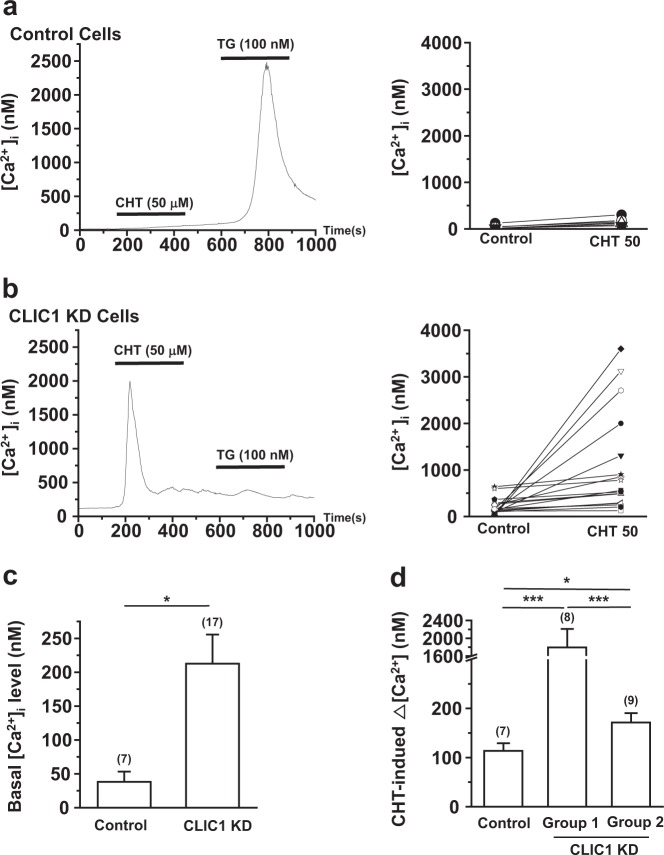
CLIC1 knockdown increased the basal Ca2+ level and augmented the effects of chelerythrine on [Ca^2+^]_i_. **a, b** Left, intracellular [Ca^2+^]_i_ recording upon exposure to chelerythrine chloride (CHT, 50 μM) in control cells (**a**) and CLIC1 KD cells (**b**). Thapsigargin (TG, 100 nM) was used as the positive control. Right, individual data from control cells (**a**) and CLIC1 KD cells (**b**). **c** Quantification of the basal [Ca^2+^]_i_ in control and CLIC1 KD A549 cells. **d** Quantification of the chelerythrine-induced Δ[Ca^2+^]_i_ in control and CLIC1 KD A549 cells, named group 1 and group 2. *P < 0.05; **P < 0.01; and ***P < 0.005 by Student’s *t*-test

Several studies have shown that alterations in intracellular Ca^2+^ can contribute to ROS generation and cellular stress^34^. To determine whether this effect occurs in CLIC1-knockdown cells, control and CLIC1 knockdown A549 cells were loaded with Fura-2 AM, and [Ca^2+^]_i_ was measured. The basal [Ca^2+^]_i_ level was significantly increased in CLIC1-knockdown A549 cells compared with that in control A549 cells (Fig. 3a, b). The estimated resting Ca^2+^ levels in control and CLIC1-knockdown A549 cells were 38.1 ± 15.2 (*n* = 7) and 212.6 ± 43.0 nM (*n* = 17), respectively (*P* *<* 0.05; Fig. 3c). Consistent with the results of a previous study^35^, chelerythrine (50 µM) had little effect on [Ca^2+^]_i_ in control A549 cells. However, in CLIC1-knockdown A549 cells, chelerythrine triggered a robust increase in the [Ca^2+^]_i_ level. Based on the peak chelerythrine-induced [Ca^2+^]_i_ increase, CLIC1- knockdown A549 cells could be divided into two groups. Even in the mild response group, the peak chelerythrine-induced [Ca^2+^]_i_ increase was higher than that in control cells (Fig. 3d). Thapsigargin (TG) was used as the positive control. These data were obtained using CLIC1-depleted A549 cells transfected with shRNA knockdown construct 1. We confirmed that chelerythrine treatment also induced a strong, transient elevation of intracellular Ca^2+^ in A549 cells with CLIC1 knockdown via shRNA knockdown construct 2 with (Supplementary Figure S3). In addition, transient small interfering RNA-mediated knockdown of CLIC1 in A549 cells resulted in strong chelerythrine-dependent Ca^2+^ elevation (Supplementary Figure S3). To verify the effects of CLIC1 knockdown on Ca^2+^ signaling, A549 cells were treated with the CLIC ion channel blocker IAA94 (50 μM) for 10~15 min, and [Ca^2+^]_i_ was measured. Similar to the data obtained with CLIC1-knockdown cells, CLIC1 inhibition by IAA94 treatment greatly increased the basal Ca^2+^ level and augmented the chelerythrine-induced Ca^2+^ increase in A549 cells (Supplementary Figure S4). Furthermore, treatment with another PKC inhibitor, bisindolylmaleimide I, did not show the same effect as chelerythrine in CLIC1-knockdown A549 cells (Supplementary Figure S5). Because PKC is the major transducer of Gq-coupled GPCR signaling, a major involvement of Gq-coupled GPCR signaling mediated by PKC can be ruled out as an explanation for the effect of CLIC1-knockdown in A549 cells. Taken together, these data suggest that CLIC1 inhibition in A549 cells increases the basal Ca^2+^ level and exacerbates the chelerythrine-induced increase in Ca^2+^.

### The increase in ROS levels in CLIC1-knockdown cells is suppressed by BAPTA-AM

**Fig. 4:**
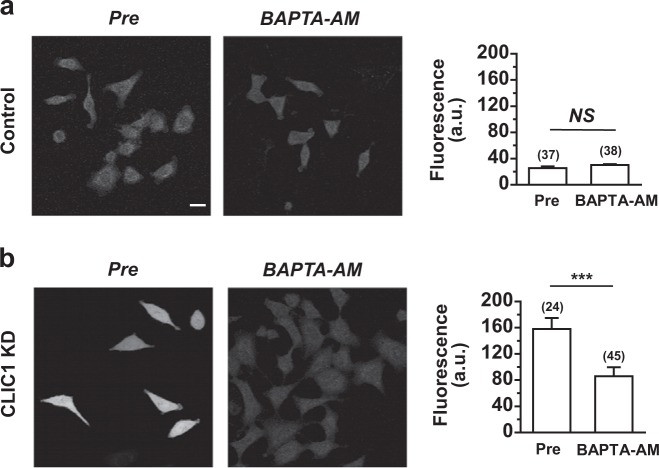
The increase in ROS levels in CLIC1-knockdown cells is rescued by BAPTA-AM. **a, b** Left, The level of ROS formation was measured in control (**a**) and CLIC1 KD A549 cells (**b**) pretreated with BAPTA-AM. Scale bars, 20 μm. Right, Summary bar graphs showing ROS levels in control and CLIC1 KD A549 cells in the absence and presence of BAPTA-AM. Means ± SEMs. ***P < 0.005 by Student’s *t*-test. *NS*, not significantly different

To determine whether intracellular Ca^2+^ plays any role in the cellular stress induced by CLIC1 knockdown, we performed studies to examine the effects of an intracellular Ca^2+^ chelator, BAPTA-AM, on ROS generation in CLIC1-knockdown A549 cells. Fura-2 AM-loaded cells were incubated with 25 µM BAPTA-AM for 30 min at 37°C and washed twice with Tyrode’s buffer. We found that the CLIC1 knockdown-induced increase in ROS generation was suppressed in the presence of BAPTA-AM (*P* *<* 0.005; Fig. 4), suggesting that an increase in [Ca^2+^]_i_ is responsible for ROS generation in CLIC1-knockdown A549 cells (Fig. 2).

### The increase in the Ca^2+^ level in CLIC1-knockdown cells is not prevented by the antioxidant Trolox

**Fig. 5:**
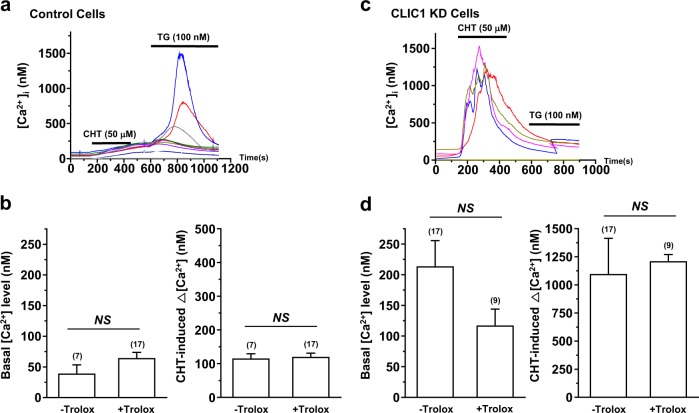
The increase in the Ca2+ level in CLIC1-knockdown cells is not prevented by Trolox. **a, c** Time-dependent measurement of [Ca^2+^]_i_ in the presence of chelerythrine chloride (CHT, 50 μM) in control cells (**a**) and CLIC1 KD cells (**c**) after pretreatment with Trolox (10 μM, 24 h). **b, d** Quantification of the basal [Ca^2+^]_i_ and chelerythrine-induced Δ[Ca^2+^]_i_ in control (**b**) and CLIC1 KD A549 cells (**d**) as shown in (**a**) and (**c**), respectively. *P*-values were determined by Student’s *t*-test, and the values shown are the means ± SEMs. *NS*, not significantly different

To examine whether ROS play a role in the increase in the basal Ca^2+^ level in CLIC1-knockdown cells, control and CLIC1-knockdown A549 cells were incubated with the widely used phenolic antioxidant Trolox (10 μM) for 24 h. As shown in Fig. 5, Trolox treatment had little effect on the basal Ca^2+^ level and chelerythrine-induced Ca^2+^ increase in both control and CLIC1-knockdown A549 cells, suggesting that ROS do not contribute significantly to the increase in the Ca^2+^ level in CLIC1-knockdown cells.

### Nifedipine inhibited the CLIC1 knockdown-induced increase in [Ca^2+^]_i_

**Fig. 6:**
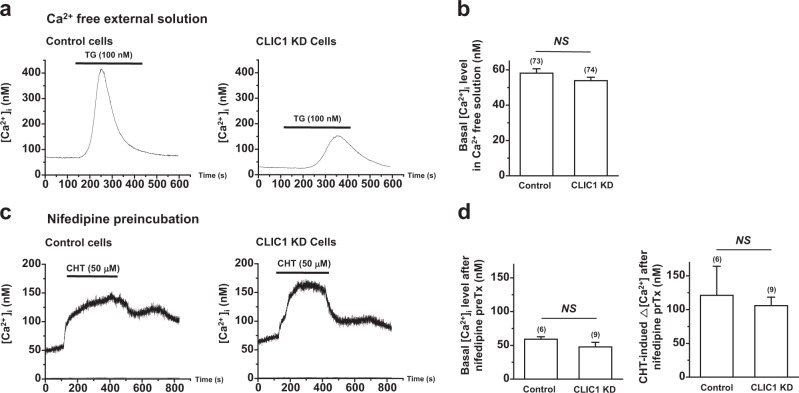
Suppression of extracellular Ca^2+^ or treatment with nifedipine inhibited the CLIC1 knockdown-induced increase in [Ca^2+^]_i_ in A549 cells. **a** Time-dependent measurement of [Ca^2+^]_i_ in control cells (left) and CLIC1 KD cells (right) after incubation in Ca^2+^-free medium for 45 min. **b** Quantification of the basal [Ca^2+^]_i_ (left) and TG-induced Δ[Ca^2+^]_i_ (right), as shown in (**a**). *P*-values were determined by Student’s *t*-test, and the data are expressed as the means ± SEMs. **c** Time-dependent measurement of [Ca^2+^]_i_ in control cells (left) and CLIC1 KD cells (right) after pretreatment with 10 μM nifedipine, an L-type Ca^2+^ channel blocker, for 24 h. **d** Histograms showing the effect of nifedipine on the basal [Ca^2+^]_i_ (left) and chelerythrine-induced Δ[Ca^2+^]_i_ (right), as shown in (**c**). *P*-values were determined by Student’s *t*-test, and the values shown are the means ± SEMs. *NS* not significantly different

We then investigated how CLIC1 regulated [Ca^2+^]_i_ in A549 cells. CLIC1 knockdown can regulate basal [Ca^2+^]_i_ either by activating constitutive Ca^2+^ entry from the extracellular environment or by promoting Ca^2+^ release from intracellular stores such as the endoplasmic reticulum. Figure 6a, b show that the suppression of extracellular Ca^2+^ reduced the basal [Ca^2+^]_i_ in CLIC1-knockdown A549 cells. This suggests that CLIC1 knockdown regulates the basal [Ca^2+^]_i_ through Ca^2+^ entry across the plasma membrane. A recent study showed that the LTCC is influenced by the concentrations of intracellular anions such as chloride^36^. Thus, we examined the possible involvement of the LTCC in the CLIC1 knockdown-induced increase in [Ca^2+^]_i_. Figure 6c, d showed that blocking the LTCC with nifedipine (10 μM, 24 h) reduced the basal Ca^2+^ level in CLIC1-knockdown A549 cells from approximately 212.6 nM to 47.8 nM, which was not significantly different from that in control A549 cells (59.0 nM, *n* = 6; *P* *>* 0.05; Fig. 6d). Furthermore, the effects of chelerythrine on [Ca^2+^]_i_ in CLIC1knockdown A549 cells were suppressed by nifedipine, and the extent of the chelerythrine-induced [Ca^2+^]_i_ increase was comparable to that in control A549 cells (Fig. 6d).

**Fig. 7:**
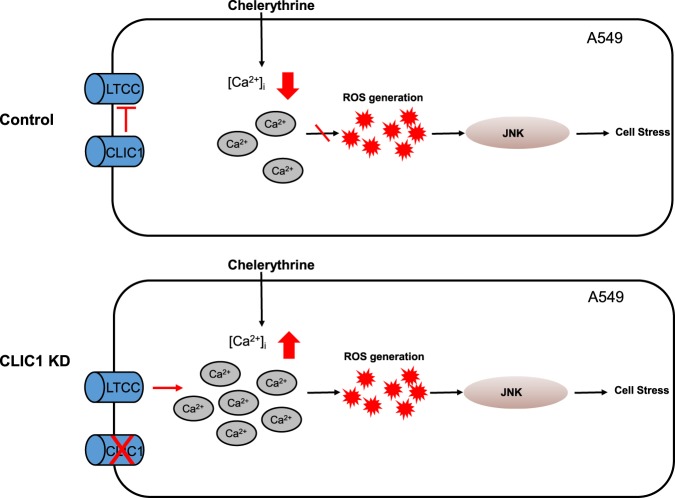
A working hypothesis. In A549 control cells, CLIC1 blocked the L-type calcium channel and diminished the effects of chelerythrine on [Ca^2+^]_i_. When CLIC1 was depleted, the intracellular calcium level was increased through the LTCC and increased calcium-enhanced ROS generation. ROS accumulation might lead to JNK phosphorylation, resulting in an increase in cellular stress

It is generally accepted that the LTCC controls intracellular Ca^2+^ in excitable cells through plasma membrane channel activity. However, in nonexcitable cells such as cancer cells, the LTCC regulates the Ca^2+^ level, often through noncanonical functions such as by regulating the expression and activity of other ion channels or proteins involved in the regulation of [Ca^2+^]_i_^37,38^. To determine whether the regulation of the Ca^2+^ signal in A549 cells by the LTCC depends on its channel activity, we performed an electrophysiological study via the patch clamp technique in the whole-cell configuration. We detected little LTCC activity in both control and CLIC1-knockdown cells even with the use of Ba^2+^ instead of Ca^2+^ to maximize the inward current conductance (data not shown). Taken together, these data suggest that CLIC1-knockdown in A549 cells causes the dysregulation of Ca^2+^ signaling, resulting in excess ROS generation and cellular stress. Based on these data, we propose a working hypothesis that CLIC1 is involved in the regulation of Ca^2+^ homeostasis through noncanonical LTCC function in A549 cells, thereby preventing excessive intracellular levels of Ca^2+^ and ROS and controlling cellular stress (Fig. 7). Therefore, CLIC1 is a key regulator of Ca^2+^ signaling in the control of cancer cell survival.

## Discussion

CLIC1 plays critical roles in processes such as apoptosis, proliferation, invasiveness, and metastasis in cancer cells, but the underlying mechanisms remain unclear. The disturbance of intracellular ROS homeostasis is reported to be a key downstream event for CLIC1 activation^28,29^, but the mechanisms by which CLIC1 regulates ROS levels are not clear, and the signaling pathways downstream of ROS disturbance need to be identified. In the present study, using electrophysiological and molecular analyses, we showed that CLIC1 knockdown induces an increase in [Ca^2+^]_i_ through the LTCC, which contributes to increased ROS levels with concomitant JNK activation. Stress-triggered JNK activation has been linked with the induction of apoptotic DNA fragmentation through H2AX phosphorylation, which accumulates at sites of DNA double-strand breaks^39^. Consistent with these observations, we found that CLIC1 deficiency exacerbates p-JNK signaling and enhances the levels of p-γH2AX, a marker for DNA double-strand breaks, in response to a putative anticancer agent, chelerythrine. The intracellular ROS measurement results revealed that CLIC1 knockdown in A549 cells upregulated ROS levels, an effect prevented by the intracellular Ca^2+^ chelator BAPTA-AM. However, the antioxidant Trolox had little effect on the basal Ca^2+^ level and the chelerythrine-induced increase in the Ca^2+^ level in both control and CLIC1-knockdown A549 cells. These data suggest that Ca^2+^ dysregulation occurs prior to ROS disturbance in these cells. Blocking the LTCC with nifedipine restored the basal Ca^2+^ level and chelerythrine-induced Ca^2+^ response in CLIC1-knockdown A549 cells to that in control cells, suggesting a role for LTCC in the increase in Ca^2+^ signaling related to CLIC1 depletion. Based on our current data, we propose that CLIC1 is critical for the control of intracellular ROS levels and the apoptotic signaling cascade by suppressing the LTCC function.

Our study demonstrates that CLIC1 regulates [Ca^2+^]_i_ through the LTCC in A549 cells because (1) the suppression of extracellular Ca^2+^ attenuated the CLIC1 knockdown-exacerbated Ca^2+^ response, and (2) nifedipine treatment blocked the increase in the basal Ca^2+^ level and chelerythrine-induced Ca^2+^ signal in CLIC1-knockdown cells. Since we detected little LTCC activity in both control and CLIC1- knockdown cells using the patch clamp technique, it is likely that the LTCC regulates the Ca^2+^ level via a noncanonical mechanism in these cells. Several nonselective cation channels such as TRPC1, TRPC3, TRPC4, and TRPC6 have been found in A549 cells^40^. Thus, the involvement of nonselective cation channels in CLIC1 knockdown-induced Ca^2+^ signaling is plausible. However, the pharmacological properties of those TRPCs exhibiting insensitivity to nifedipine^41^ exclude the possible involvement of TRPCs. Therefore, the LTCC is the most plausible candidate for increased basal Ca^2+^ levels and chelerythrine-stimulated elevation of intracellular Ca^2+^ in CLIC1-knockdown A549 cells. However, we cannot completely rule out the possible involvement of other nifedipine-insensitive nonselective cation channels. LTCC proteins are expressed in various cancers^42^ and have both canonical and noncanonical functions^37^. It has been demonstrated that LTCC proteins control Ca^2+^ homeostasis and cell migration in the HCT116 colon cancer cell line by a noncanonical mechanism that involves another channel protein, NCX1/3^38^. In addition, the LTCC can also function as a transcription factor regulating the expression of proteins involved in the regulation of [Ca^2+^]_i_ and cell migration^43,44^. Considering the broad cellular localization of CLIC1 proteins, CLIC1 might also modulate LTCC function as a transcription factor. Further studies are required to elucidate the regulatory mechanism of noncanonical LTCC function in CLIC1-knockdown A549 cells.

The reduction in the chloride channel activity in cancer cells induces an increase in the intracellular chloride level due to a reduction in chloride efflux^45^. Chloride (Cl^−^) is the most abundant transportable anion in all cells of the body, and the intracellular concentration of chloride ([Cl^−^]_i_) is regulated and maintained by a delicate functional balance between the operations of plasma membrane Cl^−^ channels and those of transporters, as well as those of local impermeant anions^46,47^. As intracellular chloride homeostasis is critical for many cell functions, including cell signaling transduction^48,49^, intracellular chloride might function as a signaling messenger to regulate LTCC directly or indirectly. In fact, previous studies showed that an increase in chloride levels can increase L-type Ca^2+^ currents^36^, possibly via two intracellular regions of the LTCC. It is also reported that the replacement of Cl^-^ with various substituting anions influences many Ca^2+^-mediated processes, including the contractility of cardiac and skeletal muscle, hormone secretion, and neurotransmitter release via the LTCC^50–54^. Thus, it is plausible that CLIC1 knockdown in A549 cells induces an increase in the intracellular level of chloride, which in turn enhances LTCC function. However, the detailed mechanisms by which intracellular chloride regulates the LTCC need to be further investigated.

Our results demonstrated that BAPTA-AM suppressed ROS production in CLIC1-knockdown A549 cells, suggesting that Ca^2+^ signaling can influence the cellular generation of ROS. Interactions between ROS and Ca^2+^ signaling can be bidirectional, wherein ROS can regulate cellular calcium signaling, while calcium signaling is essential for ROS production^55^. However, the antioxidant Trolox did not alter the basal Ca^2+^ level or chelerythrine-induced Ca^2+^ response in either control or CLIC1-knockdown A549 cells, suggesting that these Ca^2+^ signals are independent of ROS in A549 cells. These data further support the idea that the mutual interplay and crosstalk between Ca^2+^ and ROS is highly dependent on the cellular context^56^. The role of Ca^2+^ and ROS during the process of apoptosis has been explored in great depth. Ca^2+^ signals regulate ROS by modulating several ROS generation systems, including NADPH oxidases (Nox), NO synthase (NOS) and mitochondria, and the consequent Ca^2+^ and ROS surges are required for apoptosis initiation at the mitochondria-endoplasmic reticulum interface^57^. However, this interplay is altered in cancer cells, enhancing their resistance to apoptosis, but its underlying mechanisms are still unclear^57^. Taken together with the fact that the activity of JNK and p38 is regulated by intracellular Ca^2+^ as well as ROS^58,59^, our results suggest that the disturbance in intracellular Ca^2+^ signaling combined with elevated ROS levels might underlie the stronger but more transient activation of MAPK upon chelerythrine treatment in CLIC1 KD1 cells relative to that in control cells and that these surges in Ca^2+^ and ROS might lead to a cellular stress-induced response and death, implying that CLIC is important for the apoptosis resistance of A549 cells.

It appears that the regulation of ROS levels by CLIC1 is tumor cell-type specific. It has been previously shown that the inhibition of CLIC1 by IAA94 significantly suppressed ROS generation in glioblastoma cancer stem cells and LOVO cells, a human colon adenocarcinoma cell line^60^. However, CLIC1 knockdown in human esophageal squamous cell carcinoma induced apoptosis through the JNK pathway, likely reflecting excessive ROS production^61^. Similar to its effect in human esophageal squamous cell carcinoma, CLIC1 knockdown in A549 human lung cancer cells upregulated cell death and JNK activation concomitant with the elevated ROS levels. The basis for the different effects of CLIC1 inhibition in distinct cancer cells is currently unclear; however, one of the mechanisms might be the diverse regulatory crosstalk between Ca^2+^ and ROS^56^. We demonstrated that CLIC1 inhibition upregulated ROS levels by increasing intracellular Ca^2+^ through the LTCC in A549 cells. Considering that the functional expression of the Ca^2+^ signaling machinery, such as the LTCC, varies depending on cell type, downstream signaling effects, such as ROS regulation by CLIC1 inhibition, can vary in different cell types. Further studies are required to clarify these uncertainties. However, our data reveal that CLIC1 might play a critical role in apoptosis resistance, diminishing the large surges in the Ca^2+^ concentration and ROS levels, and suggest the possibility for targeting CLIC1 to control apoptosis in cancer cells.

## Supplementary information

Supplementary Figures

Supplementary Table

Supplementary Figure Legend
