# Supplementary material for: The inhibition of chloride intracellular channel 1 enhances Ca2+ and reactive oxygen species signaling in A549 human lung cancer cells
Source: Exp Mol Med. 2019 Jul 17;51(7):81. doi: 10.1038/s12276-019-0279-2 (PMC6802611; doi:10.1038/s12276-019-0279-2)

Supplementary Figure S1

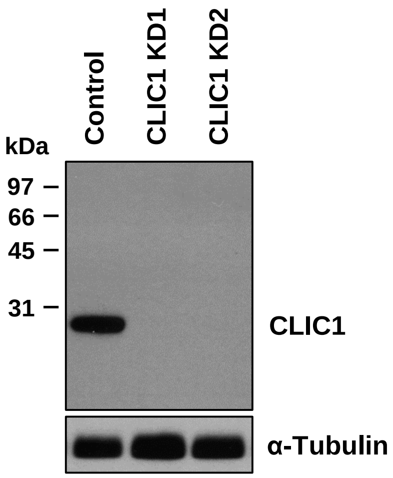

Supplementary Figure S2

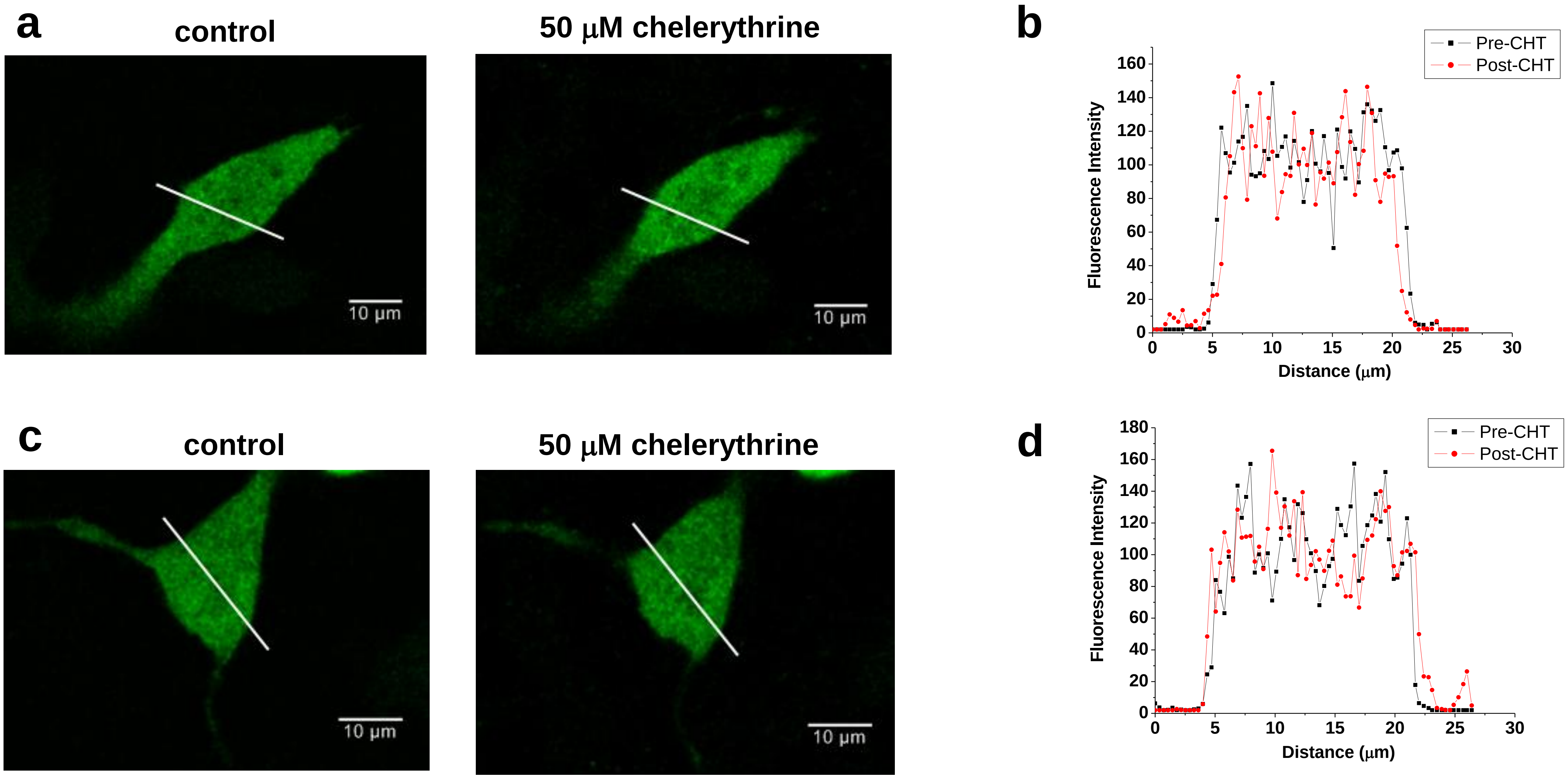

Supplementary Figure S3

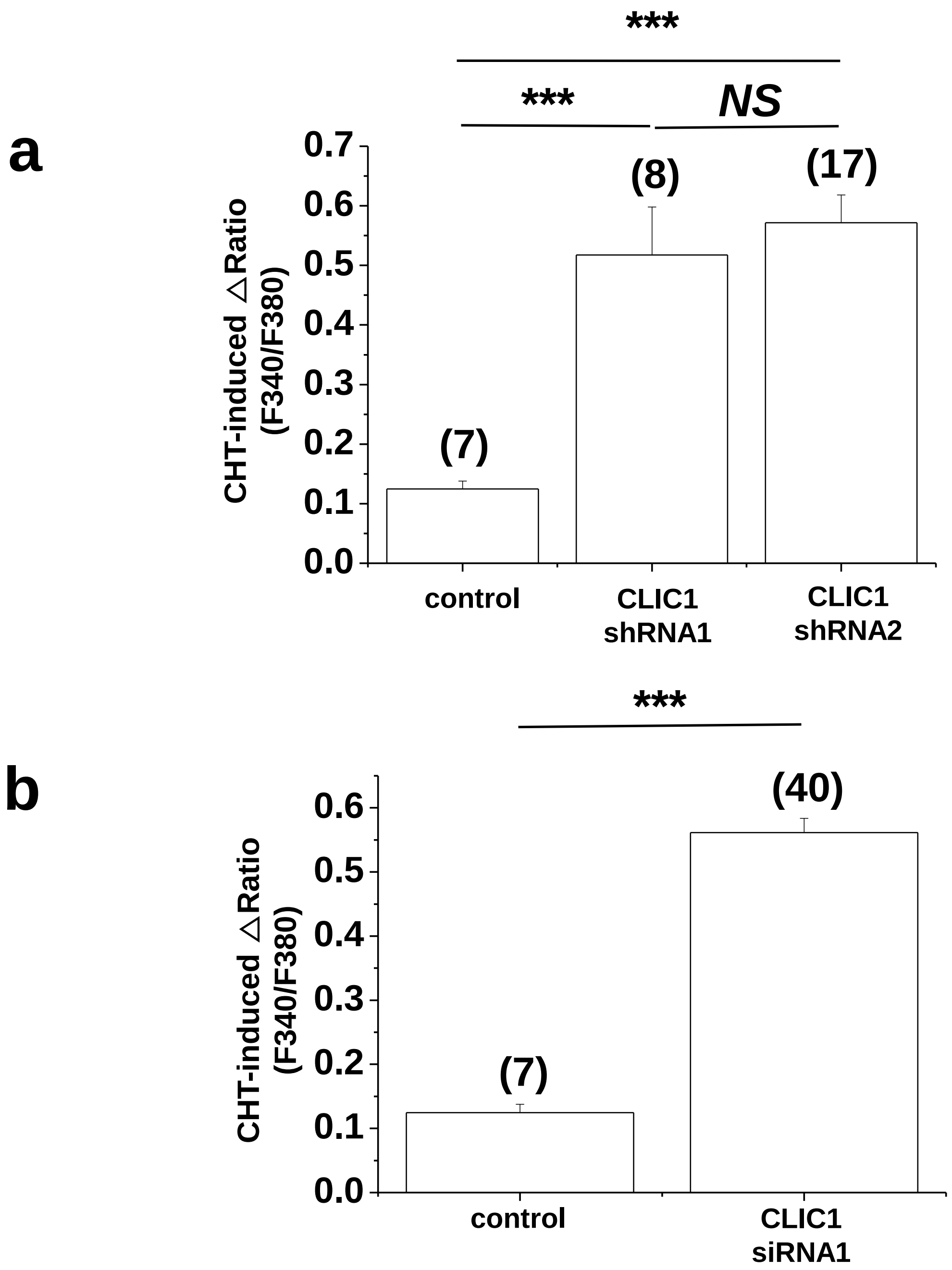

Supplementary Figure S4

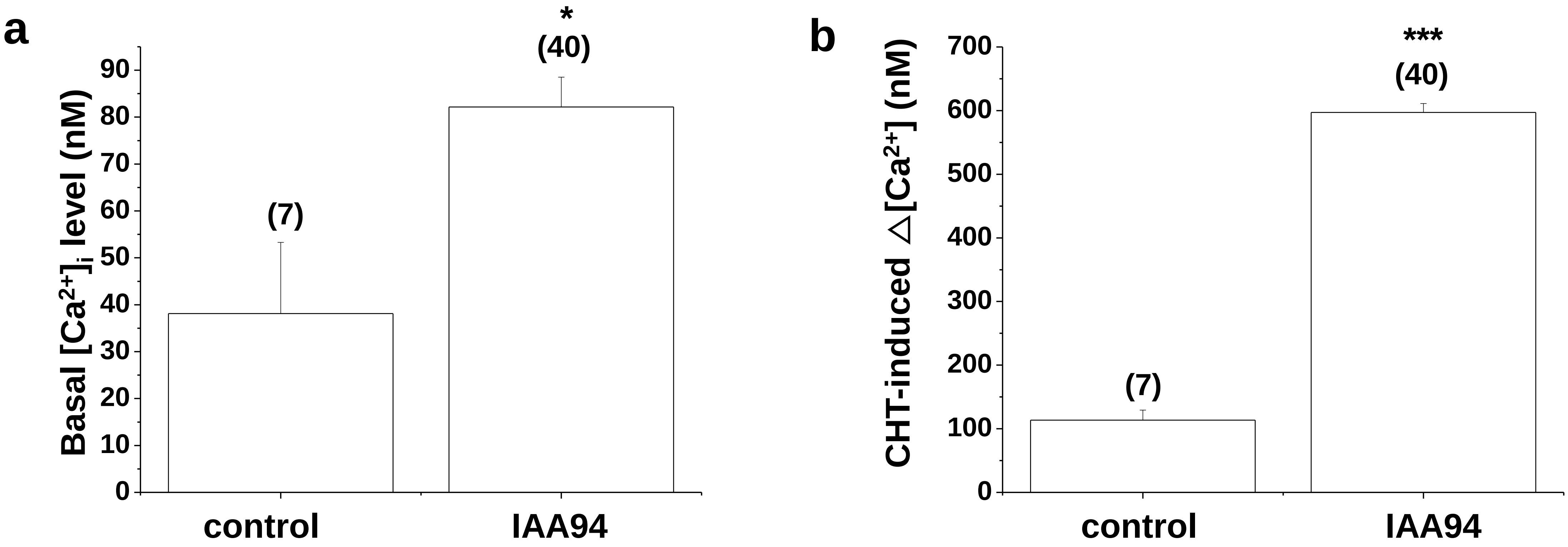

Supplementary Figure S5

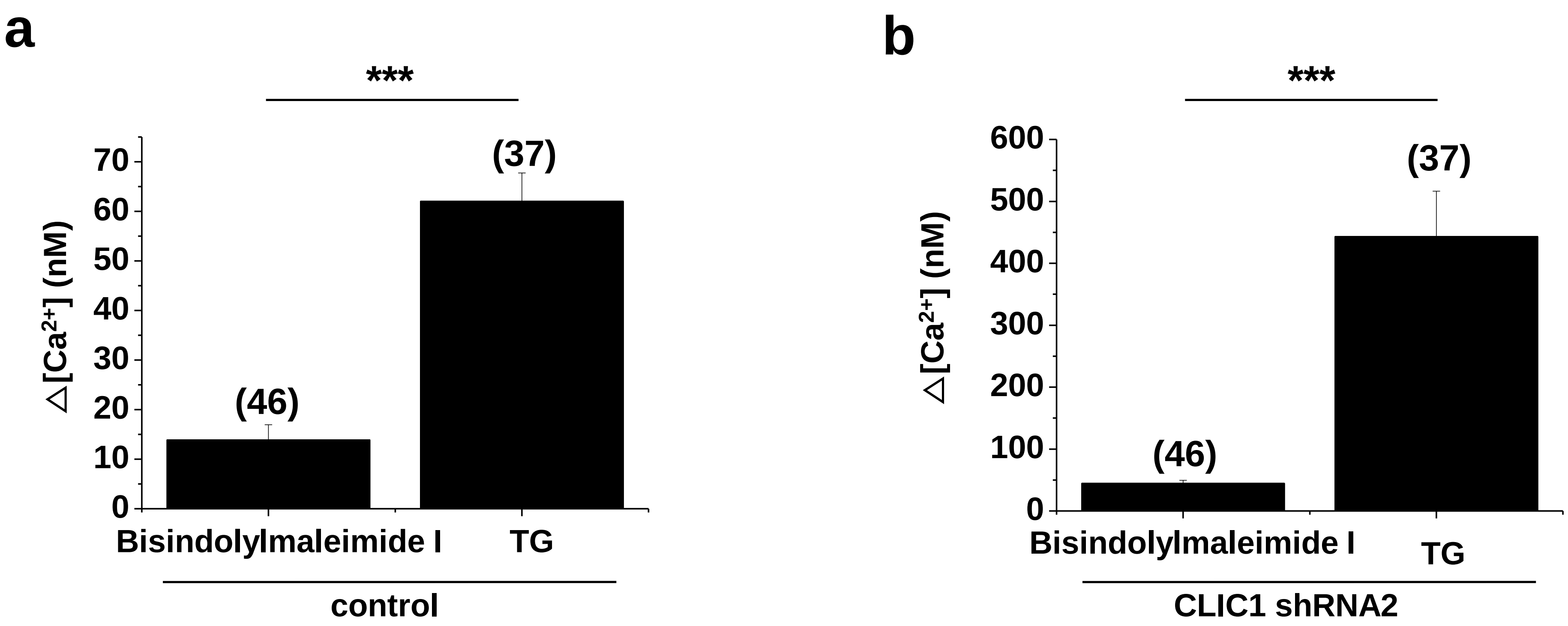

Supplement: Supplementary file 1 — Supplementary Figures [file 12276_2019_279_MOESM1_ESM.pdf]
