# Supplementary material for: The inhibition of chloride intracellular channel 1 enhances Ca2+ and reactive oxygen species signaling in A549 human lung cancer cells
Source: Exp Mol Med. 2019 Jul 17;51(7):81. doi: 10.1038/s12276-019-0279-2 (PMC6802611; doi:10.1038/s12276-019-0279-2)
Supplement: Supplementary file 2 — Supplementary Table [file 12276_2019_279_MOESM2_ESM.pdf]

	Control		CLIC1 KD1		CLIC1 KD2	
	**0 μM**	**50 μM**	**0 μM**	**50 μM**	**0 μM**	**50 μM**
**n**	417	940	340	773	513	882
