# Supplementary material for: The inhibition of chloride intracellular channel 1 enhances Ca2+ and reactive oxygen species signaling in A549 human lung cancer cells
Source: Exp Mol Med. 2019 Jul 17;51(7):81. doi: 10.1038/s12276-019-0279-2 (PMC6802611; doi:10.1038/s12276-019-0279-2)
Supplement: Supplementary file 3 — Supplementary Figure Legend [file 12276_2019_279_MOESM3_ESM.docx]

**Supplementary Figure Legends**

**Supplementary Figure S1. Expression of CLIC1 was reduced using two different shRNAs.** The protein level of CLIC1 in A549 cells with each shRNA were confirmed by immunoblot analysis. α-tubulin was used as loading control.

**Supplementary Figure S2. The CLIC1 protein localization during acute exposure to chelerythrine. a** A549 cells transfected with a CLIC1-eGFP fusion protein, shown before (left) and after (right) stimulation with 50 μM chelerythrine. Fluorescence intensities along the lines are graphically represented in (**b**). **b** Profiles of fluorescence intensity show that chelerythrine does not affect the GFP fluorescence distribution. A further example is shown in **c** & **d**. Scale bar, 10 μm.

**Supplementary Figure S3. Effects of CLIC1 knockdown on chelerythrine-induced elevation of [Ca^2+^]_i_.** The chelerythrine (50 µM)-induced increase in intracellular Ca^2+^ level was measured as the ratio (F340/F380) of Ca^2+^ dye fluorescence in A549 cells treated with CLIC1-targeted shRNAs (**a**) and CLIC1-targeted siRNA (**b**). Cells were loaded with Fura-2 AM and [Ca^2+^]_i_ was measured. Data represent means ± SEMs. *NS*, not significantly different, * *P* < 0.05, Student’s *t*-test.

**Supplementary Figure S4. IAA94 increased basal Ca^2+^ level and augmented chelerythrine effects on [Ca^2+^]_i_. a** Quantification of basal [Ca^2+^]_i_ under control condition and after 10~15 min treatment of 50 μM IAA94 in A549 cells. **b** Quantification of chelerythrine-induced Δ[Ca^2+^]_i_ under control condition and after 10~15 min treatment of 50 μM IAA94 in A549 cells. * *P* < 0.05, *** *P* < 0.005 by Student’s *t*-test.

**Supplementary Figure S5. Effects of Bisindolylmaleimide I on intracellular Ca^2+^ level in control and CLIC1-knockdown cells.** Quantification of [Ca^2+^]_i_ after 20 min treatment of 1 μM bisindolylmaleimide I in control A549 cells (**a**) and A549 cells treated with CLIC1-targeted shRNA 2 (**b**). Thapsigargin (TG, 100 nM) was used as positive control.
